# Effect of Male Cigarette Smoking on In Vitro Fertilization (IVF) Outcomes and Embryo Morphokinetic Parameters

**DOI:** 10.7759/cureus.52788

**Published:** 2024-01-23

**Authors:** Ryoma Taniguchi, Shota Hatakeyama, Shirei Ohgi, Atsushi Yanaihara

**Affiliations:** 1 Reproductive Center, Yanaihara Women's Clinic, Kamakura, JPN

**Keywords:** time-lapse imaging, smoking tobacco, male factor infertility, in vitro fertilization, embryonic development

## Abstract

This retrospective cohort study examines the association between male smoking status and embryo development in vitro. The study included non-smoking women aged under 40 years who underwent in vitro fertilization (IVF) at Yanaihara Women’s Clinic from May 2019 to May 2022, and they were divided into two groups according to the husband’s smoking status. The effect of male smoking status on IVF outcomes was compared retrospectively based on embryonic development using a time-lapse incubator. A total of 184 patients were included; 272 oocytes of 45 female non-smokers were cultured with the sperm of male smokers, and 816 oocytes of 139 female non-smokers were cultured with the sperm of male non-smokers. No significant differences were observed between male smokers and non-smokers groups with regard to fertilization and the top-quality embryo on day 3 and day 5 (p > 0.05). The male smoker group's embryos reached the early cleavage-stage parameters (time of pronuclei appearance to the five-cell stage) significantly earlier than the male non-smoker group's embryos (p < 0.05). However, no significant differences were observed between the two groups in other parameters of top-quality blastocysts (p > 0.05). It was concluded that male smoking has some differences on the timing of early embryonic events on time-lapse examination.

## Introduction

Smoking has been linked not only to a myriad of respiratory illnesses but also to a variety of health conditions. Furthermore, researchers have suggested a relationship between cigarette smoking and reproductive health. Cigarette smoke contains over 4,000 chemical components, whose mechanisms are not well known, but nicotine and carbon monoxide in particular have a negative effect [1,2]. There are many studies on the reproductive effects of male and female smoking and pregnancy outcomes. Female smoking has a negative impact on ovarian function, early menopause, and a decrease in endometrial thickness, and it affects pregnancy and childbirth, being associated with obstetrical complications, such as growth delay and premature birth [3]. In addition, male smoking decreases semen parameters and increases the sperm deoxyribonucleic acid (DNA) fragmentation index [4-6].

Although cigarette smoking has been shown to affect the outcome of assisted reproductive technology (ART), the effects of smoking on embryonic development are still controversial. Various studies have examined the effects of smoking on embryonic development [7-9]. However, the mechanisms by which cigarette smoking has a negative effect on treatment outcomes are unknown, and the effects of male smoking on fertilization and embryo development have not been determined. Traditionally, embryo quality assessment is based on morphological and developmental characteristics, such as degree of cytoplasmic fragmentation, embryo symmetry, and cleavage rate on selection, evaluated by a morphological examination of the possible pregnancy several times with a microscope. In recent years, the availability of time-lapse incubators has allowed continuous monitoring of embryo development [10]. It has become possible to perform microscopic observations in a stable culture environment in embryos in the incubator, and the initial developmental process of embryos can be observed dynamically and in greater detail compared to conventional observations [11]. Embryo evaluation by morphologic criteria at distinct time points (static evaluation) is considered the most useful non-invasive tool for selecting embryos with the highest implantation potential [10,11]. It is unclear the effect of paternal smoking on embryo development, but time-lapse cultures have suggested an effect on the relationship between male sperm morphology abnormalities and embryo morphokinetic parameters [12].

Therefore, in this study, the effects of cigarette smoking on the in vitro kinetic parameters of pre-implantation development of conventional in vitro fertilization (IVF) embryos were compared between male smokers and male non-smokes partners by a time-lapse incubator.

## Materials and methods

Patients

This was single-center, observational, retrospective study performed between May 2019 and May 2022. The protocol was approved by the ethics committee of Yanaihara Women’s Clinic, Kamakura, Kanagawa, Japan (approval no. YW19-05), and written, informed consent was obtained from all participants. Smoking habits were recorded as self-reported at the first visit to the doctor consultant before IVF cycles (i.e., the average number of cigarettes smoked daily and smoking years). The patients in this study were divided into two groups, either male smokers or non-smokers. All female partners were non-smokers in this study and both groups. The inclusion criteria were (i) all female factors with diagnosis of infertility, (ii) first oocyte retrieval cycle for IVF, (iii) treatment with the couple's gametes, and (iv) couples who consented to the study and completed the questionnaire. The exclusion criteria were (i) female age over 41 years, (ii) past smokers, (iii) second and subsequent egg collections, (iv) fresh embryo transfer cycles, and (v) use of frozen sperm and testicular sperm. The male body mass index (BMI) was calculated from self-reported weight and height, defined as weight in kilograms divided by the square of the height in meters (kg/m^2^). In addition, oocyte pick-up (OPU) was performed within 365 days of the husband’s first visit.

Ovarian stimulation protocol

In female partners, follicular stimulation was performed with a mild stimulation protocol using gonadotropin-releasing hormone (GnRH) antagonist, a GnRH-agonist short protocol, and a clomiphene protocol [13].

Sperm preparation

Semen samples were collected by masturbation, with the male partners having been requested to abstain for between three and five days. The sperm concentration and motility of all samples were assessed based on the World Health Organization (2021) guidelines [14]. Sperm selection for IVF treatment was performed by discontinuous Isolate® (FUJIFILM Irvine Scientific, CA, USA). Semen samples were layered upon a 45%/90% isolate density gradient and centrifuged at 300 g for 15 min. After centrifugation, the supernatant was removed, and 0.1 mL of HTF Medium (FUJIFILM Irvine Scientific) with 10% dextran serum supplement (FUJIFILM Irvine Scientific) was pipetted over the pellet to facilitate swimming-up of the sperm. The sample was then incubated for 20 min at 37 °C, at which point 0.1 mL of the upper layer of the media was carefully collected.

IVF and culture protocol

The insemination method was used for one to three oocytes with 150,000 motile sperm within three hours after OPU in insemination medium (Universal IVF Medium; Origio, Malov, Denmark) under OVOIL (Vitrolife, Goteborg, Sweden). Three hours after insemination, the cumulus cells and sperm were removed, and after three more hours (six hours after insemination), the oocytes were checked for the presence of the second polar body (2PB), and the oocytes with a 2PB were further cultured. Oocytes were cultured for five days with global® total® (Life global, Brussels, Belgium) and observed with an Embryo Scope+ time lapse system (Vitrolife) at 37 °C under a controlled atmosphere with low oxygen pressure (5% O_2_, 6% CO_2_). Embryos were cryopreserved on day 5 (blastocyst stage) by the vitrification method using a Vitrification Kit and Cryotop® (Kitazato, Shizuoka, Japan) and stored in liquid nitrogen. The fertilization rate was defined as the number of two pronuclei oocytes (2PN) per matured metaphase-II oocytes inseminated. Embryo morphology was evaluated on conventional scoring criteria using a day 3 cleavage-stage embryo by Veeck’s classification [15] and a day 5 blastocyst embryo by Gardner’s classification [16]. In the present study, top-quality embryos on day 3 were at least seven cells, with fragmentation of less than 10%, and day 5 top-quality blastocysts were defined as 3BB or higher in the classification.

Time-lapse monitoring of embryo morphokinetics

Time-lapse images of each day 5 top-quality blastocysts were analyzed retrospectively using the Embryo Viewer external image analysis software (Unisense Fertilitech, Aarhus, Denmark). Only good-quality blastocysts were examined in this study because the ratio of good quality blastocysts to non-good quality blastocysts is different between the two groups. Images were acquired every 10 minutes in embryo development, and embryonic events were annotated with the corresponding timing in units of hours after IVF. Annotations included the key events after insemination: the time of appearance of the two pronuclei (tPNa); time to the faded pronuclei (tPNf); the time points of embryo cleavage to the 2-, 3-, 4-, 5-, and 8-cell (t2, t3, t4, t5, and t8) stages; time to initiation of blastulation (tSB); and the formation of the blastocyst (tB). Cell cycle durations were calculated for the second cell cycles (cc2, t3-t2), the third cell cycle (cc3, t5-t3), and the times to complete the first, second, and third synchronous divisions, i.e., s1 (t2-tPNf), s2 (t4-t3), and s3 (t8-t5). The timing of each annotation was adjusted for the exact time of IVF of each embryo.

Endometrial preparation and embryo transfer

Frozen-thawed top-quality blastocysts were transferred by the natural cycle or the hormone replacement cycle. Blastocysts were thawed using the thaw kit (Kitazato) and cultured for four hours until embryo transfer. In all cases, a single embryo was transferred using a Kitazato ET catheter (Kitazato) under transabdominal ultrasound. A clinical pregnancy was confirmed by ultrasound examination of the gestational sac at six to seven weeks. Miscarriage was defined as a spontaneous pregnancy loss after an intrauterine pregnancy was detected by ultrasound. Live birth indicated a pregnancy that continued after 28 weeks of gestation with a live fetus evident.

Assessment and data analysis

All statistical analyses were performed with EZR (Saitama Medical Center, Jichi Medical University, Saitama, Japan) [17], which is a graphical user interface for R (The R Foundation for Statistical Computing, Vienna, Austria). More precisely, it is a modified version of R commander designed to add statistical functions frequently used in biostatistics. Results are expressed as the means ± standard deviation (SD) or as percentages. Proportions were compared with Fisher’s exact test, and p < 0.05 was considered significant. Mean values were compared using the Mann-Whitney U test.

## Results

A total of 184 patients were included; 272 oocytes of 45 female non-smokers were cultured with the sperm of male smokers, and 816 oocytes of 139 female non-smokers were cultured with the sperm of male non-smokers. The male smokers group smoked an average of 14.4 ± 8.7 cigarettes per day (mean ± SD) and had smoked for an average of 15.4 ± 5.2 years (mean ± SD). No significant differences were found in female age, male age, number of retrieved oocytes, semen parameters, and BMI between the male smokers and non-smokers groups (Table 1).

**Table 1:** Characteristics of patients and sperm parameters Values are presented as means ± standard deviation. BMI = body mass index

Characteristics		Smokers (n = 45 patients)	Non-smokers (n = 139 patients)	p-value
No. of cycles		45	139	-
Female age (y)		35.7±3.3	34.9±3.4	0.135
Male age (y)		36.1±7.0	36.6±5.3	0.703
Cigarettes / day		14.4±8.7	0	-
Cigarettes / y		15.4±5.2	0	-
Male BMI (kg/m^2^)		24.1±3.6	23.6±3.1	0.715
No. of retrieved oocytes (n)		5.9±4.0	6.0±5.3	0.771
Semen volume (mL)		2.7±1.4	2.7±1.2	0.923
Sperm concentration (million/mL)		77.3±42.1	73.1±43.2	0.311
Sperm motility (%)		55.6±15.0	52.1±14.9	0.267
Total motile sperm (million)		131.7±154.9	97.2±86.6	0.250

The IVF outcomes of the two groups are shown in Table 2. No significant differences were observed between the male smokers and male non-smokers groups in the fertilization rate (68.5% vs. 67.6%), the top-quality embryo rate on day 3 (61.9% vs. 63.9%), and the top-quality embryo rate on day 5 (42.9% vs. 38.7%). Furthermore, no significant difference was found between the two groups in the clinical pregnancy rate on frozen-thawed single blastocyst transfer (57.1% vs. 68.8%) (Table 2).

**Table 2:** IVF outcomes and conventional embryo scoring of male smokers and non-smokers Values are presented as means ± standard deviation. IVF = in vitro fertilization

Variable		Smokers (n = 45 patients)	Non-smokers (n = 139 patients)	p-value
Oocytes inseminated (n)		5.5±5.2	5.4±3.9	0.548
Fertilization rate (%)		68.5 (126/184)	67.6 (413/611)	0.857
Embryos cultured (n)		3.2±2.3	3.2±2.7	0.418
Top-quality embryo rate on day 3 (%)		61.9 (78/126)	63.9 (263/413)	0.752
Day 5 blastocyst formation rate (%)		79.4 (100/126)	77.5 (320/413)	0.714
Top-quality embryo rate on day 5 (%)		42.9 (54/126)	38.7 (160/413)	0.408
Clinical pregnancy rate (%)		57.1 (20/35)	68.8 (64/93)	0.220
Miscarriage rate (%)		10.0 (2/20)	20.3 (13/64)	0.504
Live birth rate (%)		85.0 (17/20)	71.9 (46/64)	0.251

The data of the time-lapse embryo morphokinetic parameters on day 5 blastocysts with only top-quality embryos are shown in Table 3. The male smoker group's embryos developed significantly earlier than those in the male non-smoker group at the tPNa (8.2±1.0 vs. 8.7±1.4 hours, p = 0.017), tPNf (22.3±2.6 vs. 23.4±2.3 hours, p = 0.008), t2 (24.7±2.7 vs. 25.9±2.3 hours, p = 0.010), t3 (34.9±3.3 vs. 36.4±3.2 hours, p = 0.004), t4 (35.8±3.3 vs. 37.4±3.1 hours, p = 0.003), and t5 (46.9±5.6 vs. 49.7±5.6 hours, p = 0.002) and had a shorter time to cc2 (t3-t2) (10.2±1.8 vs. 10.7±2.1 hours, p = 0.008). However, no significant differences were observed between the two groups in other parameters of top-quality blastocysts.

**Table 3:** Time-lapse kinetic parameters of good-quality embryo development after IVF in 103 couples, according to the male smoking status Values are presented as means ± standard deviation. t0 = insemination time; tPNa = time to pronuclei appearance; tPNf = time to pronuclear fading; t2-8 = time to 2 cells to 8 cells; tSB = time to initiation of blastulation; tB = time to formation of full blastocyst; cc2 = duration of the second cell cycle; cc3 = duration of the third cell cycle; s1 = time from tPNf to 2 cells; s2 = time division to 3 cells and subsequent division to 4 cells; s3 = time division to 5 cells and subsequent division to 8 cells

Development time from t0 (h)		Smokers (n = 54 embryos, 27 patients)	Non-smokers (n = 160 embryos, 76 patients)	p-value
tPNa		8.2±1.0	8.7±1.4	0.017
tPNf		22.3±2.6	23.4±2.3	0.008
t2		24.7±2.7	25.7±2.3	0.010
t3		34.9±3.3	36.4±3.2	0.004
t4		35.8±3.3	37.4±3.1	0.003
t5		46.9±5.6	49.7±5.6	0.002
t8		54.6±7.1	56.3±7.2	0.118
tSB		92.1±6.4	93.2±6.8	0.282
tB		100.7±6.9	102.0±6.6	0.164
tPNf-tPNa		14.1±2.5	14.7±2.5	0.066
s1(t2-tPNf)		2.4±0.5	2.3±0.5	0.189
cc2(t3-t2)		10.2±1.8	10.7±2.1	0.008
cc3(t5-t3)		11.9±3.8	13.3±3.8	0.052
s2(t4-t3)		0.8±1.5	1.0±1.6	0.340
s3(t8-t5)		7.8±6.5	6.5±6.1	0.236
tSB-t8		37.5±7.2	37.0±7.3	0.644
tB-tSB		8.6±3.3	8.8±3.7	0.818

**Figure 1:**
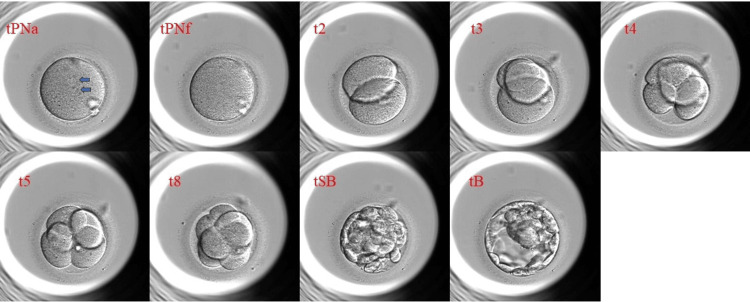
Time-lapse image examples

An example of an embryo monitored for development to the tPNa, tPNf, t2, t3, t4, t5, t8, tSB, and tB stage is shown in Figure 1.

## Discussion

This study examined the effects of embryo kinetics in IVF on the smoking status of male partners. In the present study, male smoking showed a difference in each embryo kinetic event in early development after fertilization in the time-lapse culture in IVF. However, there were no clinical effects on blastocyst formation time in late development, conventional morphological embryo evaluation, or subsequent pregnancy and miscarriage rates.

Although there have been many studies on the effects of male smoking on fertility, there have been few reports of the effects of male partner smoking on embryonic development, because the female partner’s smoking status varies [4-6]. Cigarette smoking has been reported to affect the fertilization and embryonic development of oocytes [1], and Gruber et al. reported that smoking by the female affects the fertilization ability of oocytes and the fertilization rate in intracytoplasmic sperm injection (ICSI) [18]. In addition, Freour et al. reported that the effects of smoking on the ovaries may affect embryonic development in ICSI, and delayed early development was observed in the time-lapse culture [19]. However, in the present study, there were no significant differences in the fertilization rate and in the day 3 and day 5 good embryo rates for male smokers, so it cannot be considered a negative influence. In a study by Ben-Haroush et al., the pregnancy rate of early embryo transfer in ART-treated women was decreased if the woman smoked, but the effect of passive smoking by her partner was not different from that of non-smokers [8].

In the present time-lapse monitoring results, it was observed that the early developmental events (tPNa, tPNf, t2, t3, t4 and t5) were earlier and t2 to t3 was shorter in the smoking group. Other male smoking studies have also shown earlier tPNf and t2 in early fertilization in smoking males, which may interfere with early embryonic development [20]. Although the timing of fertilization is not precisely known because of IVF, there may be a particular effect on the sperm of male smokers and some disturbance in the first mitosis, which may suggest abnormalities in chromosome distribution or spindles during the first division. However, there are some reports different to ours stating that fast early developmental kinetics are better for births, and we do not consider that these events alone could be ruled out as an effect of smoking [21].

Sperm DNA fragmentation and genetic mutations have also been suggested in the male reproductive decline in smoking, and it has been reported that tPNf, t2 is also earlier when a higher condensation defect in sperm chromatin sperm structure is found [22], so the effect of smoking male sperm on early embryonic development is undeniable. However, there was no difference in the blastocyst development time in the five-day culture in the present study. It is possible that embryonic development is preserved by the oocyte’s ability to repair the embryo [23], and it is considered unlikely that the effect of smoking on the sperm affects late development to the blastocyst.

It has been reported that sperm conditioning using density gradient centrifugation and swim-up can reduce the sperm DNA fragmentation rate in male smokers [24]. The present study does not directly show the effect of smoking in the in vivo environment or under IVF conditions, because sperm conditioning was performed, and good sperm was used for insemination. Yang et al. showed that the sperm DNA fragmentation rate was significantly higher in heavy smokers who smoked more than 20 cigarettes per day, but it was not significantly different in smokers who smoked less than 20 cigarettes per day [25]. However, in the present study, the number of cigarettes smoked per day varied among men who smoked, and qualitative sperm analysis, such as sperm DNA fragmentation, was not performed. In addition, Fosse et al. reported that the miscarriage rate increased significantly with the increase in the number of cigarettes smoked per day by the partner [26], but the present study did not evaluate the number of cigarettes smoked per day in the male smoking group.

The limitations of this study are inherent in its retrospective design, small sample size, and limited study population in a single-site analysis. They are also restricted to couples receiving ART treatment and are based on only one pre-treatment survey, thus not accounting for variations in smoking during consecutive treatment cycles.

Time-lapse imaging can provide an objective morphological assessment of embryonic development over time, and it is necessary to further consider the effects of smoking on embryonic development, which may affect the relationship between smoking and reproductive effects in men. Moreover, further research in this area is needed to understand the mechanisms of smoking’s effects on reproduction.

## Conclusions

The present study results suggest that some differences of male smoking on the timing of early embryonic events and the primary period of the fusion mechanism of the male and female genomes in IVF may hinder embryonic development in the early stages of fertilization. These effects were not observed in conventional embryo observations, and time-lapse culture revealed possible effects of male smokers on early embryonic development. Although there were no differences in morphological blastocyst development, smoking habits are considered one of the most important risk factors in the development of medical problems, and additional research is needed on the effects of smoking on fertility outcomes.
